# Isoflavone Supplementation Does Not Potentiate the Effect of Combined Exercise Training on Resting and Ambulatory Blood Pressure in Non-Obese Postmenopausal Women: A Randomized Double-Blind Controlled Trial-A Pilot Study

**DOI:** 10.3390/nu12113495

**Published:** 2020-11-13

**Authors:** Juliene G. C. Dechichi, Igor M. Mariano, Jéssica S. Giolo, Jaqueline P. Batista, Ana Luiza Amaral, Paula A. B. Ribeiro, Erick P. de Oliveira, Guilherme M. Puga

**Affiliations:** 1 Laboratory of Cardiorespiratory and Metabolic Physiology, Physical Education and Physical Therapy Department, Federal University of Uberlândia, 38400-678 Uberlândia, MG, Brazil; julienegoncalves@hotmail.com (J.G.C.D.); igormmariano@gmail.com (I.M.M.); sanjuliaogiolo@hotmail.com (J.S.G.); jaquebpontes@gmail.com (J.P.B.); anaribeiro.am@gmail.com (A.L.A.); 2 Research Center of University of Montreal Hospital Centre, Montreal, QC H2X 0A9, Canada; paulaabribeiro@gmail.com; 3 Laboratory of Nutrition, Exercise and Health (LaNES), School of Medicine, Federal University of Uberlandia (UFU), 38402-022 Uberlândia, MG, Brazil; erick_po@yahoo.com.br

**Keywords:** soy, ambulatory blood pressure monitoring, aerobic exercise, menopause, blood pressure variability

## Abstract

Physical exercise and isoflavone supplementation are potential strategies to prevent and treat cardiovascular diseases in postmenopausal women. The aim of this study was to investigate whether there are additive effects of isoflavone supplementation when associated with combined aerobic and resistance exercise on resting and ambulatory blood pressure monitoring (ABPM) and in blood pressure variability (BPV). Thirty-one non-obese postmenopausal women were randomly allocated into two groups: placebo and exercise (Placebo *n* = 19); and isoflavone supplementation (100 mg/day) and exercise (isoflavone *n* = 19). ABPM and BPV were evaluated before and after 10 weeks of moderate combined (aerobic and resistance) exercise training. Generalized Estimating Equation (GEE) with Bonferroni correction and intention-to-treat analysis was used to compare the effects of interventions on resting BP, ABPM and BPV. Combined exercise training decreased resting systolic (SBP) and diastolic blood pressure (DBP) and reduced 24 h and awake ambulatory SBP, DBP and mean blood pressure over time, with no additional effects of isoflavone supplementation. No changes were observed in sleep period, or in BPV indexes (Standard Deviation of 24 h (SD), daytime and nighttime interval (SDdn) and average real variability (ARV) in both groups. We conclude that isoflavone supplementation does not potentiate the effects of combined training on resting and ambulatorial systolic and diastolic blood pressure in non-obese postmenopausal women.

## 1. Introduction

Several physiological changes (especially in endocrine system) occur during the transition period of menopause due to the influences of the aging process, lifestyle and hypoestrogenism [1]. These factors can lead to an increase in blood pressure (BP) and body mass index, which can intensify the risk of developing cardiovascular diseases and cardiovascular events [2,3]. The prevalence of hypertension is significantly higher in individuals older than 55 years, and postmenopausal women are more affected by this disease compared with premenopausal women [2] or men at the same age [4,5].

Non-drug interventions have been sought for the benefits of reduced cost, minimal risk and proven results in reducing BP [6,7]. Among the interventions used, supplementation with phytoestrogens such as isoflavone (ISO; soy-derived compound) have been used for women in the menopause transition as an alternative to the effects of reduced estrogen production [8]. The use of ISO by postmenopausal women may have benefits in reducing cardiovascular diseases, climacteric symptoms [9] and lipid levels [10]. A meta-analysis [11] showed that isoflavone supplementation is beneficial for the reduction of systolic (SBP) and diastolic blood pressure (DBP), −5.9 mmHg and 3.3 mmHg, respectively, in hypertensive individuals; however, the results for normotensive individuals are still controversial. In addition, in rats supplemented with isoflavone for more than six months, there was an increase in endothelial nitric oxide synthase (eNOS)activity and an increase in the increase in nitric oxide (NO)bioavailability [12], which may interfere in blood flow and blood pressure responses.

Another strategy for reducing BP is exercise, which improves the bioavailability of NO [3,7], endothelial function and hemodynamic and cardiovascular parameters such as blood flow and BP [3]. In general, exercise training results in important autonomic and hemodynamic adaptations that influence the cardiovascular system, decreasing resting BP levels and improving ambulatory BP monitoring (ABPM) [13] and its variability (BPV, i.e., Ambulatory Blood Pressure Variability) [14]. These improvements may be important for the prevention and treatment of hypertension and have been shown usually in continuous moderate aerobic or resistance exercise trials [15]. However, there is a lack of studies showing ABPM and BPV responses after combined training (aerobic and resistance in the same session) protocols, especially in postmenopausal women.

We have previously shown that associating isoflavone supplementation with physical training does not modify markers of oxidative stress in postmenopausal women [16]. Further, few studies have investigated the use of isoflavone supplementation therapy associated with exercise in BP responses [17]. These results are still inconclusive, and we do not know any study that has evaluated BPV. Therefore, our hypothesis is that the supplemented group would present a greater hypotensive response to training, i.e., ISO supplementation enhances the training effect, which may be due to an increase in blood flow and endothelium-mediated vasodilation by the NO release [18,19,20]. Therefore, the aim of this study was to investigate whether there are additive effects of isoflavone supplementation, when associated with combined aerobic and resistance exercise, on resting BP, ABPM, BPV and nitrite in non-obese postmenopausal women.

## 2. Materials and Methods

### 2.1. Participants

Inclusion criteria were: age between 50–70 years, having amenorrhea of at least 12 uninterrupted months; body mass index 25–30 kg/m^2^; being able to perform treadmill and resistance exercises; no history of cardiovascular disease, stroke or acute myocardial infarction, cancer, kidney disease, diabetes or hypertension; does not use any hormone therapy or soy supplementation; non-smoker; does not use hypoglycemic agents. This study was approved by the Human Research Ethics Committee of the Federal University of Uberlândia under registration number 40622414.9.0000.5152, and all volunteers signed a consent form. The experiments followed the principles of the Declaration of Helsinki and were registered at Clinicaltrials.gov (number: NCT03008785).

This pilot randomized double-blind controlled trial was developed in two stages: baseline and after 10 weeks of ISO supplementation and training with combined exercises (aerobic and resistance). Participants were randomly allocated through an electronic draw program into two groups: exercise with placebo (PLA; *n* = 14); and exercise with isoflavone supplementation (ISO; *n* = 17). At the three weekly visits for training, the capsules were monitored and delivered by the researchers containing ISO or placebo (indistinguishable-same shape, size and color) and on the days that participants did not go to the laboratory, they were advised to take one capsule a day at the same training time. The capsules were previously packed in identical bottles and numerically organized according to the randomization schedule for each patient. The bottles were coded by a blinded researcher, so the content was unknown to researchers and participants. The ISO group ingested a dose of 100 mg of isoflavone (74.4 mg of aglycone equivalent) containing 3.3% genistein, 93.5% daidzein and 3.2% glycitein. The capsules ingested by the placebo group were non-active capsules containing 100 mg of corn starch. A blinded statistician evaluated all the results.

### 2.2. Outcomes

The primary outcome was blood pressure response assessed by the monitoring of ambulatory blood pressure. The secondary endpoints were blood nitrite concentration, heart rate variability, blood pressure variability, body composition and muscle strength.

### 2.3. Evaluation of Food Intake and Anthropometry

The food intake evaluation was performed using three 24 h dietary recalls, applied by nutritionists on non-consecutive days. The dietary data analyses were performed using a web-based program (Dietpro^®^ v5.7, Viçosa, MG, Brazil) and the United States Department of Agriculture (USDA) food composition table.

Body mass was measured using an electronic scale (Micheletti, São Paulo, SP, Brazil), and stature was measured via a stadiometer (Sanny, São Paulo, SP, Brazil). An inelastic tape (Sanny, São Paulo, SP, Brazil) measuring 0.5 cm wide was used for waist circumference measurements. Total lean body mass (LBM) and fat mass (FM) were estimated by bioelectrical impedance analysis (Biodynamics model 450c, Biodynamics, Shoreline, WA, USA).

### 2.4. Physical Activity and Exercise Intensity Evaluation

The physical activity level was assessed using the short version of the International Physical Activity Questionnaire (IPAQ) [21]. The assessment of aerobic capacity was assessed by means of a submaximal incremental test on a treadmill, adapted from Puga et al. [22]. The speed was fixed at 5.5 km/h and the intensity was increased by inclination, at 1% every two minutes until arriving at 85% of the maximum predicted heart rate (HR), or the reporting of subjective perception of effort of a score of 18 on the Borg Scale. A Cosmed Quark CPET gas analyzer (Rome, Italy) was used to record oxygen consumption (VO_2_) and carbon dioxide output (VCO_2_). The purpose of this test was to identify the ventilatory thresholds (VT1 and VT2) from the ventilatory equivalents of oxygen (VE/VO_2_ ratio) and carbon dioxide (VE/VCO_2_ ratio) [23]. The prescription of resistance exercise and strength assessment was based on the maximum repetition test (1RM) [24]. This test consisted of the workload performed with no more than one repetition in five tries, with three min of rest between tries [24].

### 2.5. Resting Blood Pressure and Ambulatory Blood Pressure Monitoring

To assess BP 24 h before and after 10 weeks of intervention, we used the Dyna Mapa + Cardius^®^ device (Cardios °Sistemas, São Paulo, SP, Brazil), programmed with measurements to be taken every 15 min during the day (7 a.m. to 11 p.m.) and every 30 min at night (11 p.m. to 7 a.m.). Together with this device, the volunteers filled out a daily record of activities such as sleep, work, food, or any event that could interfere with BP or measurements. In a standardized manner, the device was set at 7 a.m., and measurements were considered valid when 24 h of monitoring were obtained. Resting BP was measured with the same device after 15 min of rest in a sitting position, and for the analysis of the pressure curve, time 0 was adopted when the monitor was placed. SBP, DBP, mean blood pressure (MBP) and HR in periods of awake, sleep and 24 h were analyzed.

### 2.6. Blood Pressure Variability

Using ABPM data, we analyzed BPV by three different parameters [25]: standard deviation of 24 h (SD24), the time interval between consecutive readings; the mean daytime and nighttime deviations weighted by the duration of the daytime and nighttime interval (SDdn) and the average real variability (ARV) weighted by the time interval between consecutive readings.

SDdn is the mean of the daytime and nighttime standard deviations corrected for the number of hours included in each period, eliminating the influence of the day-night blood pressure difference on the BPV estimate. ARV averages the absolute differences in consecutive measurements and accounts for the order in which BP measurements are obtained [26].

### 2.7. Blood Collection and Analysis

For analysis of nitric oxide, 15 mL of blood sample was collected after an overnight fast, following a period of 72 h to five days after the last training session to eliminate any acute effects of exercise. The collection was made by an experienced nurse, and the volunteers were instructed not to exercise or to consume alcohol or caffeinated drinks in the 24 h prior to collection. After collection, the samples were centrifuged at 3000 rpm for 15 min, separated into two microtubes (1.5 mL) and stored at −80 °C until analysis. Serum NO_2_^−^ was analyzed by the total dose of it determined at 570 nm in microplate readers (Molecular Devices, Menlo Park, CA, USA), according to the protocol of Moorcroft et al. [27].

### 2.8. Exercise Program

The exercise program lasted for 10 weeks containing 30 combined sessions of aerobic and resistance training. Each session lasted 45 min, with 5 min of warm-up, 20 min of resistance and 20 min of aerobic exercise, with the order reversed in each session. Resistance training was performed in two sets of 15 repetitions via seven exercises (60% of 1RM) for large muscle groups: leg press 45°, seated low row, vertical chest press machine, seated lever fly machine, wide grip lat pull-down, swiss ball squat and abdominal crunch. Aerobic exercise was performed on a treadmill with a fixed speed of 5.5 km/h and intensity between ventilatory thresholds 1 and 2 regulated by the inclination of the treadmill. For load readjustment, after 5 weeks of training the 1RM test was performed again, and the intensity of the aerobic exercise was readjusted for a 20% increase (inclination of the treadmill).

### 2.9. Statistical Analysis

Sample calculation (*n* = 30) was performed in G-Power software v3.1 (Universität Düsseldorf, Düsseldorf, Germany) (Bicaudal; α err: 0.05; power: 0.80), adopting a significant expected difference of 22 mmHg in SBP with a standard deviation of 25 mmHg, which are the maximum ceilings [28,29]. Unpaired *t*-test was used to compare baseline characteristics between groups. BP variation over time was analyzed by area under the curve (AUC) calculated by the trapezoidal method in GraphPad Prisma Software v6 (San Diego, CA, USA) Comparison between groups and time of ABPM, AUC of BP and BPV were made by Generalized Estimates Equation (GEE) with Bonferroni correction. The GEE analyzes were performed in two ways: by protocol (including only those who completed the protocol: ISO *n* = 17; PLA *n* = 14) and by intention to treat (including those who did not complete the study: ISO *n* = 19; PLA *n* = 19) using the last-observation-carried-forward method. NO_2_^−^ were made by ANOVA two way. A *p*-value of <0.05 was used for statistical significance, and all statistical analyses were performed using SPSS software v20.0 (IBM, New York, NY, USA).

## 3. Results

**Figure 1:**
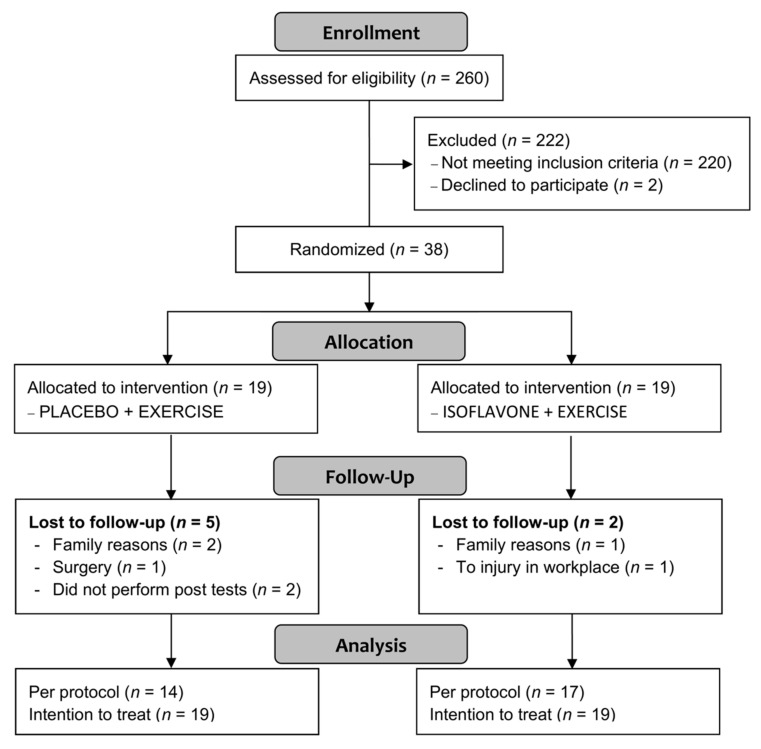
Flowchart of study participants.

A total of 260 postmenopausal women, aged 50–70, were recruited via traditional media (TV, radio and posters) from January to December 2015. From a total of 260 responders, 38 non-obese fulfilled the inclusion criteria and were randomized; 33 completed the 10 weeks of training and 31 performed post-tests (Figure 1).

Characteristics of participants are listed in Table 1. There was no difference between the PLA and ISO groups in age, time after menopause, body mass index and physical activity level measured by International Physical Activity Questionnaire (IPAQ) and metabolic equivalent of task (MET). Values of maximum strength performed in 1RM and anthropometric measurements are listed in Table 2. There was a time effect in all resistance exercises but no interaction between groups and time effects, so both groups increased their strength as measured by 1RM test. There was also no difference between groups and time in body mass, BMI, total lean mass, waist circumference and fat mass.

The volunteers trained at an intensity (treadmill inclination) of 5% ± 5%, a rate of perceived exertion of 16 ± 2 and a heart rate (HR) of 158 ± 16 bpm. Although there was no dietary control, the analysis of dietary data did not show significant differences between groups or over time (data published by Giolo et al. [16]). No changes were observed in the consumption of macronutrients (carbohydrates, proteins and lipids) and branched chain amino acids (total BCAA, isoleucine, leucine and valine).

**Figure 2:**
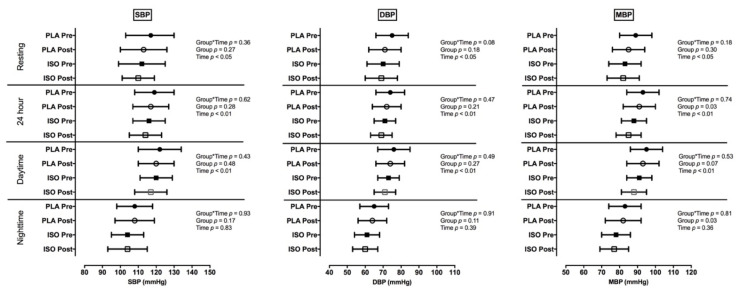
Intention-to-treat analysis of resting and ambulatory blood bressure monitoring AMBP results during 24 h, night-time and daytime periods (mean ± SD). PLA: placebo and exercise group; ISO: isoflavone and exercise group; SBP: systolic blood pressure; DBP: diastolic blood pressure; MBP: mean blood pressure; Pre: Measures pre interventions; Post: Measures post interventions.

Figure 2 shows intention-to-treat analysis of the BP measures obtained during resting and ABPM in the awake, sleep and 24 h periods. There were no interaction effects (group*time) in daytime (power analysis: Intention to treat: Daytime: SBP = 0.738; DBP = 0.576; MBP = 0.887; nighttime: SBP = 0.984; DBP = 0.952; MBP = 0.986; 24 h periods: SBP = 0.504; DBP = 0.292; MBP = 0.766). On the other hand, SBP, DBP and MBP decreased (*p* < 0.01) in both groups in 24 h and awake times. SBP and DBP, at rest also decreased after the intervention in both groups. No interaction was found in SBP, DBP or MBP AUCs over 24 h or in HR during these periods of the day (data non-shown). These results using per protocol analysis is in the Supplementary Figure S1.

Table 3 shows intention-to-treat analysis of BPV results. No changes in ARV (Power analysis ARV: SBP = 0.959; DBP = 0.948; MBP = 0.948), SD24h (Power analysis SD24h: SBP = 0.697; DBP = 0.602; MBP = 0.640) and SDdn (Power analysis SDdn: SBP = 0.748; DBP = 0.828; MBP = 0.799.) were found in time or interaction (group*time) effects. These results using per protocol analysis is in the Supplementary Table S1.

**Figure 3:**
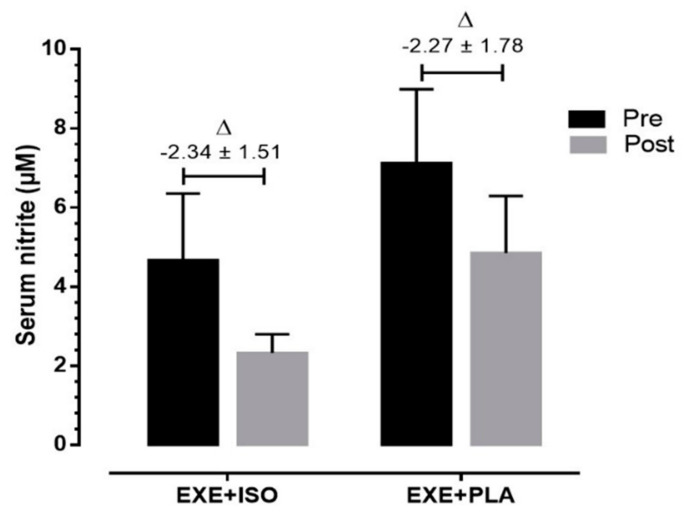
Values of serum NO_2_^−^ variation in both placebo (PLA) and isoflavone (ISO) groups. EXE: exercise; Δ: final minus baseline values. ANOVA two way was used to compare groups and time. Student *t*-test was used to compare delta values.

Figure 3 shows serum NO_2_^−^ variation in both PLA and isoflavone ISO groups. There was no interaction (group*time) or difference in group or time (Power analysis of serum NO_2_^−^ = 0.594).

## 4. Discussion

Our study analyzed the effects of isoflavone supplementation associated with combined aerobic and resistance training on resting BP, ABPM, serum NO_2_^−^ and BPV in non-obese postmenopausal women. Our main findings were that no additive effects of isoflavone supplementation on exercise training-mediated hemodynamic responses in non-obese menopause women were found. However, the exercise training improved the resting BP, and 24 h ambulatory BP, but not BPV. Moreover, our volunteers improved their muscle strength and aerobic capacity and no differences were found between anthropometric characteristics for time or group interactions.

Acute and chronic exercises for postmenopausal women have been shown to have beneficial effects on fat and lean mass and also for the prevention and treatment of metabolic and cardiovascular diseases [30]. Changes in body composition are common after menopause, such as reduced lean mass and bone mass, reduced muscle strength and changes in fat deposition with increased visceral fat [31] due to aging and also reduced levels of estrogen [32]. Our study showed that the combination of aerobic and resistance training in the same session was effective in improving strength in both groups, playing an important part in the treatment in decreasing and/or delaying these changes. In our study, these improvements are probably due to exercise training since we were not able to demonstrate any additive effect of isoflavone, as demonstrated in previous studies [33,34].

Exercise training can improve cardiovascular parameters such as resting BP responses in both hypertensive and normotensive patients, including postmenopausal women [35]. These results seem to be greater in aerobic training when compared to resistance training [15]. However, evidence shows that combined exercise training shows a less pronounced reduction in resting BP compared with aerobic or resistance training, but this can be due to the lack of studies using these modalities as comparative groups [15]. Our data showed that combined exercise training could improve both rest and ambulatory SBP, DBP and MBP with no additive effect of isoflavone supplementation, a result which is not well established in normotensive postmenopausal women due to the lack of relevant studies.

ABPM is an important method to demonstrate the behavior of BP [36], which can provide 24 h monitoring with data referring to awake and sleep periods. Moreover, these data allow the analysis of BPV with reduced discomfort during daily routine activities [37]. Some studies show that high values of both ABPM and BPV during 24 h are associated with a higher risk for cardiovascular diseases such as hypertension and cardiovascular events, making this an important parameter for cardiovascular health monitoring [14]. The use of BPV has increased due to the possibility of analysis of fluctuating values over time, which is additional information compared to in-office measurements, and considering milder variations as lower risk [14].

In this study, we did not find differences for any BPV indexes, which corroborates with several studies of healthy men and women in the same age group [38,39,40]. However, in populations with cardiometabolic dysfunctions, the results appear to be more promising [41,42,43]. It is worth mentioning that most studies use only aerobic training [38,39,40], few use dynamic [44] or isometric [45] resistance training, and we are not aware of studies with combined training. In addition, not only the type but the intensity of exercise training seems to be related to variations in BPV, and its route of effect seems to be through adaptations of vascular endothelium and smooth muscle [46]. In view of this, exercise seems to play an important role in modulating BP variations in individuals with dysfunctions, but has little or no influence on healthy subjects undergoing mild to moderate intensity training for a few weeks [42,47].

Importantly, vascular responses are mediated by multiple factors. It has been demonstrated that exercise training improves the bioavailability of NO [48] improving endothelial function, and hemodynamic and cardiovascular parameters such as BP [3,22]. A previous study showed increased serum NO_2_^−^ concentration in postmenopausal women, but the intervention consisted of aerobic training alone, with a longer intervention, and was followed by decreased in resting BP [29,49]. Nevertheless, we were not the first group to find no effect on BP responses after exercise training with ISO supplementation [50,51,52]. We believe that training characteristics [53], population [54] and dietary control [55] could play an important role in these responses.

Its chemical similarity to estrogen allows that, although with less affinity than the hormone, isoflavone acts through the stimulating mechanisms involved in the activity of nitric oxide synthase, increasing the production of NO and the control of calcium channels [56], mechanisms that are directly linked to the control of BP. This effect of isoflavone was found in the study carried out by Walker et al., in which the main components of isoflavone (genistein and daidzein) were administered to analyze the vasodilator effect when compared to estrogen. It was found that the acute administration of genistein in the brachial artery produces vasodilation in the forearm vasculature similar to that caused by estrogen, and that doses used compared to that consumed by Asian populations [57].

Chronic physical exercise [7] and isoflavone [12] stimulate the production of NO, which is directly linked to the reduction of BP and vasodilation. Because vascular health is reduced with age and hypoestrogenism, a condition of postmenopausal women [58], this may establish a physiological limit for the activation or production of NO by this route, so that the two isolated interventions can be beneficial; but when associated, they do not result in their potentiated effects, but are neutralized. The post-menopausal normotensive population evaluated in our study may show an absence of effects after isoflavone supplementation, in which the vasculature and absorption are not as efficient for obtaining high results as in pre-menopause; but despite these effects, isoflavone may compensate for the effects of the disease and for hypoestrogenism as found in hypertensive postmenopausal women.

A meta-analysis showed that the duration of supplementation can also interfere with the results, being more effective when interventions last for more than three months in hypertensive patients [59]. Although the total amount of isoflavone used in our study is within the effective range (25–375 mg), the intervention duration may have been insufficient to demonstrate effects. However, our results corroborate those found by Carmignani et al. [60], who used similar amounts of isoflavone (90 mg isoflavone containing 26.5 mg of aglycone: ~8 mg of daidzein, 15 mg of genistein and 3.5 mg of glycitein) and found no effect on BP, even with 16 weeks of intervention. It is noteworthy that this study shows methodological differences, not only in the duration, but mainly in a protocol without exercise intervention, and uses different amounts of types of isoflavone, which limits the comparison of findings between the studies.

In addition, the total amount of isoflavone seems to be less important than the specific amount of its compounds, genistein being the main type of isoflavone. The amount of isoflavone (100 mg) and its compounds (3.3% genistein, 93.5% daidzein and 3.2% glycitein) used in our study may have interfered with the findings obtained, since studies that demonstrated beneficial effects of isoflavone on vasomotor symptoms [61], BP [62] and arterial compliance [63] used amounts of genistein greater than 15 mg. In contrast, studies that used less than 10 mg did not present reduction of symptoms [64].

The main limitation of the study was the sample size, which was estimated using an expected change of 22 mmHg of SBP, resulting in a relatively small number of volunteers per group. Some variables also have a low power of analysis, making it difficult to generalize these results. Another limitation of our study was that, despite the greater amount of daidzein used, it may have failed to convert to equol (a non-steroidal estrogen) and so may not have allowed us to achieve better results. For conversion to take place, a specific intestinal microorganism, present in 30% to 50% of individuals, is essential to achieve the benefits obtained with supplementation [65]. In addition, other factors act as influencers on the complete action of phytoestrogens, affecting their bioavailability and their biological effects, such as a diet rich in carbohydrates that increases fermentation, diseases related to the intestinal tract, parasitosis and use of antibiotics [66], which were not checked in our volunteers. In addition, although our study was designed to assess the ISO additive effect, it has the limitation of not verifying the isolated effect in the ISO supplementation group. The 10-week intervention period may have been insufficient to demonstrate potentiated responses in BP in non-obese postmenopausal women. Thus, additional studies evaluating the possible effects of ISO and its mediators associated with exercise on hypotension mechanisms are needed, as the current evidence seems to demonstrate a limited impact on healthy subjects.

## 5. Conclusions

The addition of isoflavone supplementation to combined aerobic plus resistance training does not change training-mediated responses in resting and ambulatory blood pressure and serum nitrite levels. However, since our results suggest that the combined training improved resting and ambulatory blood pressure, it should be considered for interventions focused on reverse menopausal effects in non-obese women.

## Figures and Tables

**Table 1:** Characteristics of participants (*n* = 31).

Clinical Characteristics	PLA (*n* = 14)	ISO (*n* = 17)	*p*
Age (years)	53 ± 5	56 ± 5	0.08
Time after menopause (years)	4 ± 4	7 ± 5	0.14
Body mass index (kg/m^2^)	26.9 ± 0.7	26.2 ± 0.2	0.51
Physical activity level-MET (min/week)	954 ± 990	1268 ± 869	0.35

PLA: placebo and exercise; ISO: isoflavone and exercise; MET: metabolic equivalent of task. Unpaired *t*-test was used to compare groups. Data were described on average e standard deviation.

**Table 2:** Comparison of clinical, anthropometric and strength characteristics at baseline and after 10 weeks combined exercise training moments between groups using placebo + exercise (PLA − *n* = 14) and isoflavone + exercise (ISO − *n* = 17) interventions.

	Baseline Mean ± Standard Error	10 Weeks Mean ± Standard Error	*p* (Time)	*p* (Groups)	*p* (Group*Time)	Change Mean (95% IC)
Body Mass (kg)						
PLA	64.7 ± 2.1	65.7 ± 2.3	0.12	0.91	0.46	1.1 (−1.2 to 3.3)
ISO	65.3 ± 2.0	65.7 ± 2.0				0.4 (−0.6 to 1.4)
Body Mass Index (kg/m^2^)						
PLA	26.9 ± 0.7	25.1 ± 1.9	0.28	0.54	0.66	−1.80 (−6.53 to 2.92)
ISO	26.2 ± 0.8	26.5 ± 0.8				0.24 (−0.21 to 0.69)
Total Lean Mass (kg)						
PLA	41.7 ± 1.0	42.6 ± 1.0	0.04	0.99	0.02	0.9 (0.5 to 1.6)
ISO	44.2 ± 0.9	42.2 ± 0.8				−0.6 (−0.5 to 0.4)
Fat Mass (kg)						
PLA	23.8 ± 1.3	23.0 ± 1.4	0.22	0.89	0.13	−0.8 (−1.7 to 0.1)
ISO	23.6 ± 1.5	23.7 ± 1.6				0.1 (−0.6 to 0.8)
Waist Circumference (cm)						
PLA	92.8 ± 1.9	91.9 ± 2.0	0.04	0.96	0.49	0.9 (−2.9 to 0.9)
ISO	93.2 ± 2.3	91.2 ± 1.8				−1.9 (−4.0 to 0.6)
1RM leg press (kg)						
PLA	164.9 ± 8.0	249.7 ± 10.1	<0.01	0.30	0.54	84.8 (69.5 to 100.1)
ISO	154.8 ± 10.3	232.6 ± 12.0				78.5 (65.4 to 91.5)
1RM bench press (kg)						
PLA	27.1 ± 1.0	37.2 ± 1.0	<0.01	0.01	0.06	10.1 (8.2 to 12.0)
ISO	25.0 ± 1.2	32.2 ± 1.3				7.2 (4.8 to 9.5)
1RM lat pull down (kg)						
PLA	31.3 ± 1.7	41.2 ± 2.5	<0.01	0.30	0.28	9.9 (6.0 to 13.7)
ISO	30.1 ± 0.9	37.7 ± 1.5				7.4 (5.3 to 9.6)
1RM peck deck (kg)						
PLA	20.0 ± 1.3	31.1 ± 1.4	<0.01	0.26	0.20	11.1 (9.0 to 13.2)
ISO	19.3 ± 1.0	28.5 ± 1.1				9.2 (7.2 to 11.3)
1RM seated row (kg)						
PLA	57.7 ± 2.1	74.5 ± 1.5	<0.01	0.41	0.36	16.8 (13.7 to 19.9)
ISO	56.5 ± 2.8	71.1 ± 2.1				14.6 (11.0 to 18.2)

PLA: placebo and exercise; ISO: isoflavone and exercise; WT6: 6−minute walk test; 1RM: 1 maximum repetition test. GEE with Bonferroni correction was used to compare groups, time and interaction (group*time). Data were described on average e standard error.

**Table 3:** Intention-to-treat analysis of ambulatory blood pressure variability evaluated before (baseline) and after 10 weeks of exercise training in both placebo + exercise (PLA) and Isoflavone + exercise (ISO) groups.

Blood Pressure Variability	Baseline Mean ± SD	10 Weeks Mean ± SD	*p* (Time)	*p* (Groups)	*p* (Group*Time)	Change Mean (95% IC)
ARV SBP (mmHg/min)						
PLA	9.42 ± 0.47	9.45 ± 0.59	0.68	0.06	0.63	0.03 (−0.90 to 0.95)
ISO	10.96 ± 0.62	10.63 ± 0.60				−0.32 (−1.40 to 0.76)
ARV DBP (mmHg/min)						
PLA	6.67 ± 0.32	6.90 ± 0.31	0.83	0.07	0.24	0.23 (−0.35 to 0.81)
ISO	7.77 ± 0.36	7.44 ± 0.45				−0.33 (−1.10 to 0.40)
ARV MBP (mmHg/min)						
PLA	6.46 ± 0.28	6.63 ± 0.29	0.50	0.07	0.94	0.17 (−0.36 to 0.70)
ISO	7.24 ± 0.32	7.38 ± 0.44				0.14 (−0.57 to 0.85)
SD24h SBP (mmHg)						
PLA	12.50 ± 0.98	12.03 ± 0.76	0.61	0.21	0.74	−0.47 (−2.14 to 1.19)
ISO	13.62 ± 0.64	13.53 ± 0.86				−0.10 (−1.53 to 1.33)
SD24h DBP (mmHg)						
PLA	9.56 ± 0.46	9.70 ± 0.48	0.97	0.26	0.69	0.14 (−0.91 to 1.18)
ISO	10.44 ± 0.53	10.28 ± 0.64				−0.16 (−1.23 to 0.90)
SD24h MBP (mmHg)						
PLA	9.61 ± 0.63	9.50 ± 0.54	0.96	0.24	0.83	−1.12 (−1.42 to 1.18)
ISO	10.31 ± 0.48	10.39 ± 0.62				0.07 (−1.06 to 1.21)
SDdn SBP (mmHg)						
PLA	10.52 ± 0.66	10.32 ± 0.58	0.84	0.18	0.73	−0.21 (−1.32 to 0.90)
ISO	11.41 ± 0.54	11.47 ± 0.62				0.06 (−0.96 to 1.08)
SDdn DBP (mmHg)						
PLA	7.69 ± 0.35	8.33 ± 0.47	0.41	0.14	0.29	0.63 (−0.31 to 1.58)
ISO	8.79 ± 0.42	8.71 ± 0.46				−0.08 (−1.01 to 0.84)
SDdn MBP (mmHg)						
PLA	7.70 ± 0.40	8.00 ± 0.44	0.42	0.15	0.96	0.29 (−0.71 to 1.30)
ISO	8.38 ± 0.33	8.63 ± 0.45				0.26 (−0.61 to 1.12)

SBP: systolic blood pressure; DBP: diastolic blood pressure; MBP: mean blood pressure; PLA: placebo group; ISO: isoflavone group; ARV: average real variability; SDdn: standard deviation of daytime and nighttime; SD24h: standard deviation for 24 h. Generalized Estimating Equation (GEE) with Bonferroni correction and intention-to-treat analysis was used to compare groups, time and interaction (group*time). Data were described on average e standard deviation.

## Supplementary Materials

The following are available online at https://www.mdpi.com/2072-6643/12/11/3495/s1, Figure S1: Per Protocol analysis of resting and AMBP results during 24 h, nigthttime and daytime periods (mean ± SD). PLA: placebo and exercise group (*n* = 14); ISO: isoflavone and exercise group (*n* = 17); SBP: systolic blood pressure; DBP: diastolic blood pressure; MBP: mean blood pressure; Pre: Measures pre interventions; Prost: Measures post interventions, Table S1: Per Protocol analysis of ambulatory blood pressure variability evaluated before (baseline) and after 10 weeks of exercise training in both placebo + exercise (PLA *n* = 14) and Isoflavone + exercise (ISO *n* = 17) groups.
