# Supplementary material for: Isoflavone Supplementation Does Not Potentiate the Effect of Combined Exercise Training on Resting and Ambulatory Blood Pressure in Non-Obese Postmenopausal Women: A Randomized Double-Blind Controlled Trial-A Pilot Study
Source: Nutrients. 2020 Nov 13;12(11):3495. doi: 10.3390/nu12113495 (PMC7697944; doi:10.3390/nu12113495)
Supplement: Supplementary file 1 [file nutrients-12-03495-s001.pdf]

Table S1. Per Protocol analysis of ambulatory blood pressure variability evaluated before (baseline) and after 10 weeks of exercise training in both placebo + exercise (PLA *n* = 14) and Isoflavone + exercise (ISO *n* = 17) groups.

	Baseline Mean ± SEM	10 weeks Mean ± SEM	p (Time)	p (Groups)	p (Group*Time)	Change Mean (95% IC)
			**ARV SBP (mmHg/min)**			
PLA	9.08 ± 0.32	9.12 ± 0.59	0.81	0.02	0.64	0.1 (−1.4 to 1.6)
ISO	10.72 ± 0.62	10.37 ± 0.59				−0.3 (−1.5 to 0.8)
			**ARV DBP (mmHg/min)**			
PLA	6.65 ± 0.33	6.97 ± 0.31	0.95	0.13	0.19	0.4 (−0.4 to 1.2)
ISO	7.60 ± 0.34	7.25 ± 0.45				−0.3 (−1.1 to 0.4)
			**ARV MBP (mmHg/min)**			
PLA	6.36 ± 0.31	6.59 ± 0.29	0.44	0.08	0.83	0.3 (−0.4 to 0.9)
ISO	7.04 ± 0.27	7.18 ± 0.43				0.1 (−0.6 to 0.9)
			**SD24h SBP (mmHg)**			
PLA	12.32 ± 1.12	11.66 ± 0.71	0.66	0.18	0.75	−0.6 (−3.6 to 2.3)
ISO	13.08 ± 0.56	12.97 ± 0.73				−0.1 (−1.6 to 1.4)
			**SD24h DBP (mmHg)**			
PLA	9.69 ± 0.55	9.88 ± 0.57	0.95	0.62	0.77	1.1 (−1.4 to 1.7)
ISO	10.13 ± 0.47	9.96 ± 0.60				−0.2 (−1.3 to 0.9)
			**SD24h MBP (mmHg)**			
PLA	9.78 ± 0.74	9.62 ± 0.60	0.89	0.53	0.79	−0.2 (−2.2 to 1.7)
ISO	10.03 ± 0.43	10.10 ± 0.56				0.1 (−1.1 to 1.3)
			**SDdn SBP (mmHg)**			
PLA	10.30 ± 0.67	10.01 ± 0.51	0.84	0.08	0.76	−0.3 (−2.3 to 1.7)
ISO	11.04 ± 0.45	11.10 ± 0.60				0.1 (−1.0 to 1.1)
			**SDdn DBP (mmHg)**			
PLA	7.68 ± 0.33	8.55 ± 0.51	0.32	0.26	0.23	1.0 (−0.4 to 2.2)
ISO	8.67 ± 0.44	8.58 ± 0.48				0.1 (−1.1 to 0.9)
			**SDdn MBP (mmHg)**			
PLA	7.68 ± 0.41	8.08 ± 0.48	0.42	0.25	0.88	0.4 (−1.0 to 1.8)
ISO	8.23 ± 0.33	8.50 ± 0.47				0.8 (−1.0 to 1.2)

SBP: systolic blood pressure; DBP: diastolic blood pressure; MBP: mean blood pressure; PLA: placebo group; ISO: isoflavone group; ARV: average real variability; SDdn: standard deviation of daytime and nighttime; SD24h: standard deviation for 24 h. Generalized Estimating Equation (GEE) with Bonferroni correction was used to compare groups, time and interaction (group*time). Data were described on average e standard error.

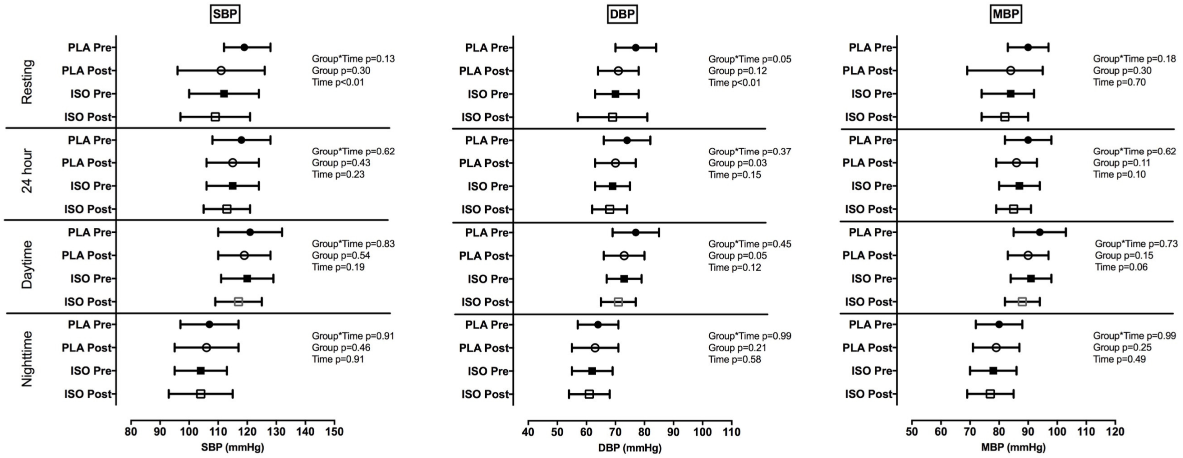

Figure S1. Per Protocol analysis of resting and AMBP results during 24h, nigthttime and daytime periods (mean ± SD). PLA: placebo and exercise group (*n* = 14); ISO: isoflavone and exercise group (n=17); SBP: systolic blood pressure; DBP: diastolic blood pressure; MBP: mean blood pressure; Pre: Measures pre interventions; Prost: Measures post interventions.
